# Kyste hydatique de la rate: à propos d'un cas

**DOI:** 10.11604/pamj.2013.14.92.2343

**Published:** 2013-03-09

**Authors:** Touré Alpha Oumar, Kâ Ibrahima, Sarr Ibrahima Sitor, Cissé Mamadou, Konaté Ibrahima, Kâ Ousmane, Dieng Madieng, Dia Abdarahmane, Touré Cheikh Tidiane

**Affiliations:** 1 Service de Chirurgie Générale, Hôpital Aristide Le Dantec, Dakar, Senegal

**Keywords:** Kyste hydatique, rate, splénectomie, hydatid cyst, spleen, splenectomy

## Abstract

Le kyste hydatique de la rate est une affection rare en Afrique sub-saharienne. Il arrive en 3^ème^ position après celui du foie et du poumon. Le traitement est essentiellement chirurgical en dehors d'un traitement médical ayant prouvé son efficacité. Nous rapportons le 1^er^ cas pris en charge au Service de Chirurgie Générale du CHU Le Dantec à Dakar. Il s'agit d'un patient de 33 ans, originaire de Mauritanie, sans antécédent particulier qui nous a été adressé pour prise en charge d'un kyste splénique de découverte fortuite à l’échographie lors d'un bilan de routine avec sérologie hydatique positive à Eccinochoccus granulosa. L'examen clinique était sans particularité. La biologie retrouvait une thrombopénie à 88000 plaquettes/mm^3^. Le scanner montrait des kystes spléniques avec calcifications occupant plus de la moitié du parenchyme splénique. Il a bénéficié d'une splénectomie totale dont les suites ont été simples. Aucune particularité n’était notée après un suivi d'un an. Le kyste hydatique splénique est rare. Le traitement est chirurgical qu'il soit radical ou conservateur avec de bons résultats.

## Introduction

Le kyste hydatique (KH) est une affection endémique notamment dans les pays méditerranéens mais rare dans les pays sub-sahariens [1]. Les localisations les plus fréquentes sont le foie, et le poumon. Le KH splénique représenterait environ 4% des localisations abdominales de l'hydatidose [2]. Il s'agit donc d'une affection rare qui peut poser un problème d'ordre diagnostique et thérapeutique surtout dans les pays non endémiques. Nous rapportons le premier cas de KH splénique pris en charge au Service de Chirurgie Générale de l'Hôpital Aristide Le Dantec de Dakar.

## Patient et observation

Il s'agit d'un patient de 33 ans, sans antécédents pathologiques particuliers, initialement reçu au service de médecine interne pour la prise en charge d'une thrombopénie a 84000 éléments/mm^3^ découverte de manière fortuite lors d'un bilan de routine. Une échographie abdominale avait objectivé une lésion splénique hypo-échogène d'allure kystique. La sérologie hydatique était en faveur d'une échinococcose (Echinococcus granulosa). C'est dans ce contexte que le patient nous a été adressé pour prise en charge chirurgicale. A l'examen, l’état général était conservé avec des constantes hémodynamiques stables (Température à 37.2°C, Tension artérielle à 130/80 mmHg, Fréquence respiratoire à 18 cycles par minute, Fréquence cardiaque à 65 battements par minute). L'examen abdominal retrouvait un abdomen souple sans masse palpable.

**Figure 1:**
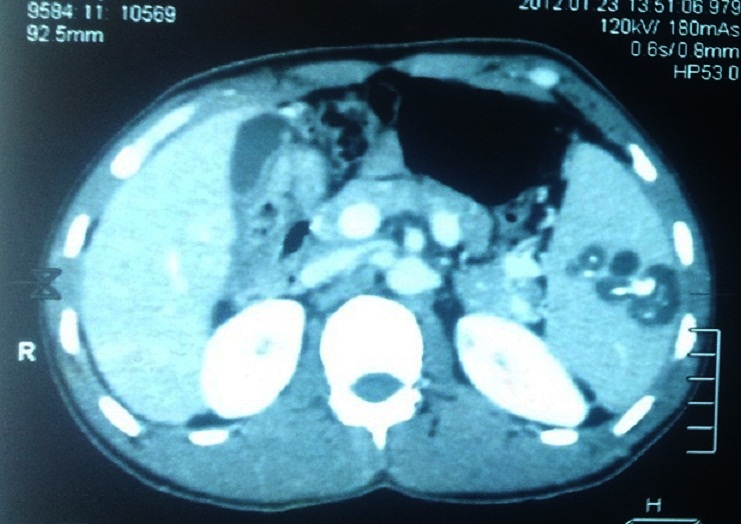
Kyste de la rate avec calcifications intra-kystiques (coupe coronale)

**Figure 2:**
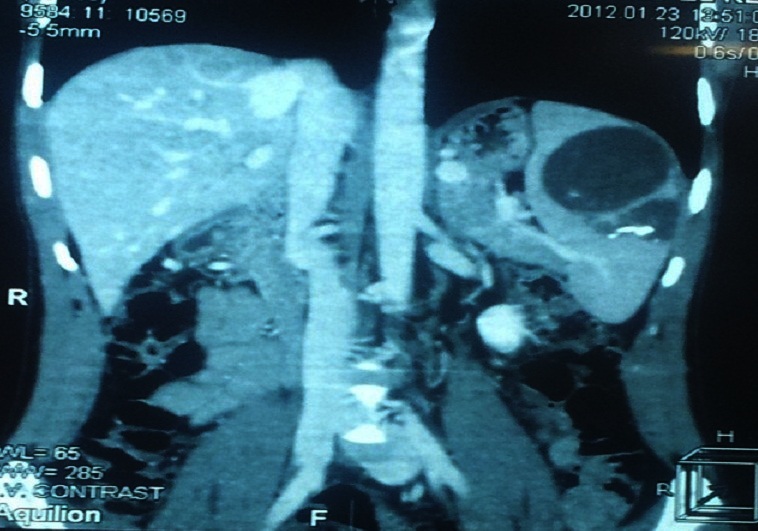
Kyste hydatique splénique (Reconstruction)

Le bilan pré-thérapeutique biologique (Numération formule sanguine et crase sanguine) montrait une thrombopénie à 88000 plaquettes/mm^3^. Le scanner abdominal avait permis d'objectiver des lésions liquidiennes spléniques contenant des calcifications faisant évoquer des kystes hydatiques (Figure 1, Figure 2). Une laparotomie réalisée a permis de retrouver à l'exploration des adhérences spléno-pariétales et omento- spléniques, un foie d'allure cirrhotique à surface micro-nodulaire et une rate augmentée de volume polykystique. Le patient a bénéficié d'une splénectomie. Les suites opératoires ont été simples. Une vaccination anti- pneumococcique et anti-méningococcique A et C ont été réalisées en post-opératoire. Le patient a été mis en exéat au 6ème jour post-opératoire. Aucune particularité n’était notée après un suivi d'un an.

## Discussion

L'hydatidose est une helminthiase provoquée par le développement chez l'homme de la forme larvaire de l'Echinococcus granulosus. L'hôte définitif est le plus souvent le chien [3]. L'hôte intermédiaire, contaminé par voie digestive, est le plus souvent le mouton et accidentellement l'homme [3]. L'embryon traverse alors la paroi intestinale, gagne le foie, par voie portale pour s'y localiser ou pour gagner les poumons par voie cave, puis n'importe quel autre organe par l'intermédiaire de la circulation systémique. Le KH est ainsi localisé le plus souvent dans le foie puis dans les poumons [3]. La localisation splénique vient en 3ème position. D'autres voies d'atteinte splénique ont été évoquées: l'atteinte par contiguïté (trans-pariétale gastrique ou colique), la voie lymphatique et la voie veineuse porto-splénique rétrograde [1, 3]. Il s'agit d'une affection rare en dehors des zones d'endémie. En Afrique, elle est surtout rencontrée au Maghreb d'où est originaire notre patient, notamment la Tunisie (15.1 cas/100000 habitants/an) [4]. Les KH spléniques atteignent surtout l'adulte de 30 à 40 ans avec une légère prédominance féminine [1].

Les motifs de consultation les plus fréquents sont la douleur, la constatation d'une masse de l'hypochondre gauche et la découverte fortuite comme pour notre patient. Le KH splénque peut aussi être découvert lors de complications telles que l'abcédation, la fissuration avec anaphylaxie et la rupture dans la plèvre, l'estomac, le colon ou à la peau [1, 3–5]. L’échographie, le scanner et l'imagerie par résonance magnétique de l'abdomen sont les examens les plus utiles au diagnostic, montrant des calcifications kystiques, des vésicules filles ou des septa intra-kystiques [1, 3–6]. Combinés à la sérologie hydatique, ces examens d'imagerie permettent la confirmation diagnostique du KH splénique [1, 4]. Le scanner de notre patient retrouvait des kystes avec calcifications intra-kystiques de la rate; la sérologie hydatique était également positive confirmant ainsi le diagnostic de KH splénique. Il peut, néanmoins, se poser quelques difficultés diagnostiques avec les autres kystes non parasitaires de la rate du fait de leur présentation clinique et radiologique similaires [2, 7].

Le traitement médical à base d'imidazolés est prescrit pour les formes multi-viscérales même si les résultats sont insuffisants [1]. Le traitement des KH spléniques est essentiellement chirurgical [1, 2, 4]. La splénectomie totale a l'avantage de supprimer l'organe parasité et d’éviter les récidives secondaires, comme pour notre patient. Elle peut être difficile en raison d'adhérences kysto- viscérales. La résection du dôme saillant a l'avantage d’être une intervention bénigne, peu hémorragique, presque toujours réalisable, dès lors que le KH est accessible à la surface de la rate. Par contre, elle laisse du périkyste en place pouvant être siège de cavité résiduelle et d'infection postopératoire [1]. La voie d'abord dépend de la localisation du ou des KH spléniques, et de l'association à d'autres localisations kystiques. L'abord laparoscopique est réalisable pour presque tous les cas, avec de bons résultats à court et à long terme [1, 4, 8].

## Conclusion

Le KH splénique est une affection rare hors de zones d'endémie d'eccinochoccose. Le diagnostic est confirmé par l'association d'une sérologie hydatique positive et d'images spécifiques à l'imagerie. Le traitement est essentiellement chirurgical avec, notamment la splénectomie totale. La laparoscopie permettrait de diminuer la morbidité post-opératoire.
